# Contribution of DNA Metabarcoding to the Environmental Fungal Assessments in Hospitals

**DOI:** 10.1007/s00248-025-02626-w

**Published:** 2025-11-24

**Authors:** Laura García-Gutiérrez, Emilia Mellado, Pedro M. Martin-Sanchez

**Affiliations:** 1 Grupo Microbiología Ambiental y Patrimonio Cultural, Instituto de Recursos Naturales y Agrobiología de Sevilla (IRNAS), Consejo Superior de Investigaciones Científicas (CSIC), Avda. Reina Mercedes 10, 41012 Seville, Spain; 2 Laboratorio de Referencia e Investigación en Micología, Centro Nacional de Microbiología, Instituto de Salud Carlos III (ISCIII), Ctra. de Pozuelo 28, 28222 Majadahonda, Madrid Spain; 3 Centro de investigación Biomédica en Red – Enfermedades Infecciosas (CIBERINFEC-CB21/13/00105): ISCIII, Majadahonda, Madrid Spain

**Keywords:** Indoor fungi, Mycobiome, Buildings, Healthcare settings, Nosocomial infections, Environmental DNA

## Abstract

Hospitals are particularly sensitive environments where immunosuppressed patients might acquire invasive fungal infections. Therefore, it is necessary to carry out periodical environmental microbiological assessments that evaluate the fungal bioburden in air and surfaces from different hospital zones. Current microbiological monitoring protocols at healthcare settings are mostly based on cultivation, while environmental DNA (eDNA) assessments are still scarce and should be further evaluated. To fill this gap, this study combines a large sampling scheme, comprising > 200 samples (air, surface, dust and soil) collected from four zones at three Spanish hospitals in two campaigns (winter and autumn), with two eDNA approaches (DNA metabarcoding and quantitative PCR) to characterize the hospital mycobiomes (diversity, community composition and airborne load), compared to a parallel culture-dependent study. DNA metabarcoding revealed a much more comprehensive inventory of hospital fungi compared to culturing; however, both approaches found similar dominant taxa including a variety of potentially opportunistic human pathogens. Hospital mycobiomes were affiliated to 4 phyla (mostly *Ascomycota* and *Basidiomycota*), 35 classes, 114 orders, 305 families, 643 genera and 535 species. The dominant genera, in both air and surfaces from the three hospitals, were *Cladosporium*, *Alternaria*, *Aureobasidium*, *Penicillium*, *Neodidymelliopsis*, *Aspergillus*, *Pseudopithomyces* and *Stemphylium*. The yeasts *Candida* and *Clavispora* were particularly abundant on high-touch surfaces indoors. The most important explanatory factors for the variance in community composition were the hospital and zone where samples were collected, the type of sample and the sampling campaign. DNA metabarcoding can assist hospital managers by providing an in-depth characterization of the baseline hospital mycobiome during normal operating conditions, as well as identifying and controlling community imbalances and associated health risks under demanding situations such as construction works or reported clinical outbreaks.

**Supplementary Information:**

The online version contains supplementary material available at 10.1007/s00248-025-02626-w.

## Introduction

Invasive fungal infections (IFI) are systemic diseases with high morbidity and mortality rates caused by the growth of filamentous fungi and yeasts in deep tissues [1]. In the last years, the prevalence of IFI has increased worldwide due to the global rise of two factors: the number of immunocompromised patients suffering other diseases such as cancer, diabetes and AIDS [2] and the antifungal drug resistance reports in some of the most relevant IFI-causing fungi, typically opportunistic species belonging to the genera *Aspergillus* and *Candida* [3–5]*.* Besides, the recent coronavirus disease pandemic has been associated with an increase of IFI such as aspergillosis, mucormycosis and candidaemia [6–8].

In this context, hospitals, where many immunosuppressed patients (e.g. cancer, autoimmune diseases, organ transplantation and severe burn injury) receive care and treatments, are especially vulnerable environments for acquiring both superficial and invasive fungal infections through the inhalation of aerosols or contact with contaminated surfaces. Therefore, it is necessary to carry out environmental microbiological monitoring programmes that evaluate the fungal bioburden in air and surfaces of hospitals. An appropriate environmental monitoring plan can detect fungal contamination sources, minimize the exposure of the most vulnerable patients and ultimately prevent the nosocomial IFI [9, 10].

Current microbiological assessment protocols in hospitals are mostly based on culture-dependent approaches on environmental samples collected by volumetric air impactors, surface swabs/sponges and contact plates [10, 11]. These methods have the advantage of focusing on viable and metabolically active organisms, which might infect the exposed people. However, it is well-recognized that the vast majority of microorganisms are not cultivable using standard media and incubation conditions due to different factors such as variation of microbial growth rate, presence of antimicrobial compounds or stresses induced by sampling (e.g. media desiccation, rebound effect and turbulence) [12].

To overcome these limitations, environmental DNA (eDNA) approaches, such as real-time quantitative polymerase chain reaction (qPCR) and next-generation sequencing (NGS) targeting PCR-amplified genetic markers (aka “DNA metabarcoding”) or whole genomes (aka “shotgun metagenomics”), have been increasingly implemented in the last decade to improve our knowledge on the microbiology of the built environment, mostly on bacteria but also on fungi (i.e. the indoor mycobiome), in residential buildings, kindergartens, schools and occupational settings [13, 14]. In particular, qPCR has been widely used to detect and quantify many indoor fungal species, genera or higher taxa [15], and DNA metabarcoding has become a key tool for surveying environmental mycobiomes regardless of the types of sample/matrix and their origin [16]. However, eDNA-based studies in healthcare settings, where many immunosuppressed occupants may be exposed to opportunistic pathogens, are still scarce and should be further evaluated. These molecular approaches can be effective and fast alternatives for studying the fungal burden in hospitals and its influence on human health, describing the whole community and/or tracking specific opportunistic pathogens [17].

Previous studies have implemented qPCR protocols for monitoring different taxa, mostly *Aspergillus* sections/species, in the hospital environment [17–19]. To date, only a few studies, with limited numbers of samples, have addressed the environmental mycobiome in healthcare facilities by shotgun metagenomics or DNA metabarcoding. Yooseph et al. [20] did a first approximation using metagenomics on a few air samples from different environments including a medical centre (one sample), with a poor taxonomical description of eukaryotes. Similarly, King et al. [18] conducted a longitudinal metagenomics study on air samples from eight locations at a hospital in San Diego, CA, USA, reporting very limited fungal information, basically, an increase of *Eurotiales* (i.e. *Aspergillus* and *Penicillium*) during some weeks of this study. Tong et al. [21] also used metagenomics to compare the airborne mycobiomes from four areas (four air samples) of a hospital in Beijing (China), where *Aspergillus* and *A. fumigatus* were found to be the most prevalent genus and species, respectively. More recently, Habibi et al. [22], Núñez and García [23] and Chen et al. [24] applied DNA metabarcoding to study the airborne hospital mycobiomes in Kuwait (2 hospitals, 4–6 samples per hospital), Spain (one hospital, 4 sampling points) and China (5 hospitals, 2 samples per hospital), respectively.

Our previous study characterized the cultivable fungal communities in three Spanish hospitals, covering numerous (> 200) air and surface samples collected from four different zones (three indoors and one outdoors) in two sampling campaigns (winter and autumn) [25]. Such a comprehensive sampling scheme allowed us to carry out simultaneous sampling for eDNA analyses and to compare the results coming from different methodologies. Considering the exposed background, the three specific aims of this study are (i) to improve the knowledge about the environmental hospital mycobiomes (diversity and community composition) using eDNA approaches, mostly DNA metabarcoding complemented by qPCR, (ii) to identify the main driving factors of the indoor hospital mycobiome and (iii) to evaluate the pros and cons of eDNA methods compared to culture-based methods for environmental microbiological assessment in healthcare settings.

## Methods

### Sampling

Since the sampling scheme has been fully described in a previous publication [25], we provide a condensed version here. Two sampling campaigns were performed in three tertiary-care hospitals from Spain (Virgen del Rocío in Seville—“VR”, 37°21′42″N 5°58′50″W; La Fe in Valencia—“LF”, 39°26′37″N 0°22′32″W; and Severo Ochoa in Leganes, Madrid—“SO”, 40°19′14″N 3°46′09″W) in winter (January–February) and autumn (September–October) 2023. Four hospital zones with different exposure/protection degrees were compared in each hospital: (“OUT”) pedestrian paths outside the buildings, (“IA”) admission halls and waiting rooms with mechanical ventilation systems and relatively high occupancy, (“IB”) empty and clean regular patient rooms with mechanical ventilation systems and (“IC”) clean intensive care units (ICU) with controlled ventilation using high efficiency particulate air (HEPA) filters and restrictions of access to people and using protective clothes. Three specific sampling points (i.e. outdoor spaces, halls, rooms or ICUs) were studied for each hospital zone. To homogenize the conditions in IB and IC zones, we selected patient rooms and ICU beds that were previously unoccupied and cleaned.

A total of 233 samples were collected from the hospital environments: 72 air samples (3 m^3^ volume; from 2 campaigns × 3 hospitals × 4 zones × 3 points), collected at 70–150 cm height using the cyclonic Coriolis µ sampler (Bertin Technologies, Montigny-le-Bretonneux, France) and sterile cones containing 15 ml PBS buffer with 0.005% Triton X; 54 surface samples from the intake ventilation grids (“vent”; from 2 campaigns × 3 hospitals × 3 indoor zones × 3 points; a unique ventilation grid was swabbed for each indoor sampling point); 54 high-touch surface samples (“HTS”; i.e. combined samples from bedrails, mattress zippers, bed tables, drip stands and monitors at IB and IC zones, or counters, seats, touch screens and vending machines at IA zones; from 2 campaigns × 3 hospitals × 3 indoor zones × 3 points); 18 settled dust from outdoor surfaces such as window sills and railings (“dust”; from 2 campaigns × 3 hospitals × 1 outdoor zone × 3 points; swabbing variable surface area until collecting enough material with the sponges); 18 soil samples from gardens or potted plants outside hospitals (“soil”; from 2 campaigns × 3 hospitals × 1 outdoor zone × 3 points; a 50-ml tube per sample); and 17 additional samples (14 from a previous pilot sampling campaign in VR and 3 extra samples collected during the winter campaign). The latter additional samples were excluded from the final statistical comparisons on homogeneous replicated groups from two campaigns (winter and autumn). All surface samples (“vent”, “HTS” and “dust”) were collected using sterile 3 M sponge-sticks soaked with neutralizing buffer (3 M, St. Paul, MN, USA). Coriolis cones were transported in dry ice to the laboratory, while sponge-sticks and soil samples were kept on ice considering their parallel use for DNA- and culture-based analyses.

Environmental indoor air quality (IAQ) variables such as six fractions of particles, air temperature (AT), wet-bulb temperature (WB), dew point temperature (DP) and relative humidity (RH), measured in situ by a PC200 particle counter (Trotec GmbH, Heinsberg, Germany), and airborne fungal colony forming units (CFU) on Sabouraud dextrose chloramphenicol agar (SDCA) and dichloran rose bengal chloramphenicol agar (DRBC) media [25], were included in this study.

### DNA Extraction

Once in the labs, the samples were processed in biosafety cabinets. Content of Coriolis cones was filtrated using sterile 25-mm cellulose acetate filters with a 0.2-µm pore diameter (Sartorius Stedim biotech GmbH, Göttingen, Germany). Surface sponges were processed as follows: adding 20 ml PBS buffer with 0.05% Tween 20 to the bags, a 2-min hand massage, orbital shaking at 180 rpm for 10 min and centrifugation at 3500 rpm for 15 min. Air filters, pelleted cells from surfaces and soil (~ 500 mg) samples were transferred to the PowerBead Pro tubes from the DNeasy PowerSoil Pro Kit (Qiagen GmbH, Hilden, Germany), and their DNA was extracted following the corresponding manufacturer’s instructions. Considering the expected very low DNA/RNA yields from air and surface samples, and the uncertain RNA integrity during transportation, we discarded other molecular methods to assess viability such as RNA-based analysis and propidium monoazide (PMA) pretreatment.

### Fungal DNA Metabarcoding

Fungal libraries were constructed employing a two-step PCR protocol. For the first PCR, duplicate PCR reactions contained DNA extracts from air (2 and 4 µl), surface (1 and 2 µl of five times diluted extracts) and soil (10–20 ng) samples, dNTPs (200 µM), primers (0.25 µM), 0.4 units of Q5 High-Fidelity DNA Polymerase (New England Biolabs Inc., Ipswich, MA, USA) and its PCR buffer (1 ×) in a final volume of 25 µl. Primers include Illumina adaptor sequence attached to the fungi-specific sequences at the internal transcribed spacer (ITS) regions, described by Tedersoo et al. [26] and Wurzbacher et al. [27]: ITS3-Mix2 (forward; 5′-ACACTCTTTCCCTACACGACGCTCTTCCGATCT-NNNNNN-CAWCGATGAAGAACGCAG-3′) and an equimolar mixture of the reverse primers ITS4-cwmix1 (5′-GTGACTGGAGTTCAGACGTGTGCTCTTCCGATCT-NNNNNN-TCCTCCGCTTAYTGATATGC-3′) and ITS4-cwmix2 (reverse; 5′-GTGACTGGAGTTCAGACGTGTGCTCTTCCGATCT-NNNNNN-TCCTCCGCTTATTRATATGC-3′). The first PCR program included an initial denaturation step of 98 °C for 1 min, followed by 30 cycles of 20 s at 98 °C, 30 s at 55 °C, 1 min at 72 °C and a final extension of 7 min at 72 °C. The resulting amplicons were approximately 350–450 bp in length, covering approximately 130 bp of the 5.8S rRNA gene and the entire ITS2 region. Duplicate reactions were subsequently pooled and purified using AMPure XP beads (Beckman Coulter Inc., Brea, CA, USA). The second PCR reactions, in 25-µl volume, included the same polymerase, buffer and dNTPs, 2 µl of the first PCR purified product as template and the sample-specific primer combinations that include 8-bp barcodes (i5 or i7 index) and complete Thruplex adapters for Illumina sequencing: (forward) 5′-AATGATACGGCGACCACCGAGATCTACAC-[i5 index]-ACACTCTTTCCCTACACGACG-3′ and (reverse) 5′-CAAGCAGAAGACGGCATACGAGAT-[i7 index]-GTGACTGGAGTTCAGACGTGTGCTCTTCCGATCT-3′. The second PCR program included an initial denaturation at 98 °C for 30 s, followed by 15 cycles of 10 s at 98 °C, 30 s at 66 °C, and 72 °C, with a final extension step of 2 min at 72 °C. Amplified DNA fragments were purified with AMPure XP beads and subsequently quantified with Qubit dsDNA HS Assay Kit (Invitrogen—Life Technologies, Eugene, OR, USA).

PCR-positive samples (167 of 233 environmental samples: 72%), excluding 25 “vent” and 40 “HTS” failed samples, were pooled equimolarly together with technical replicates (9 duplicated samples), negative samples (4 unused filters and sponges), extraction negatives (7), PCR negatives (3) and mock samples (3 with an equimolar DNA mixture from four fungal isolates from the hospital environment, *Papiliotrema*, *Cercospora*, *Peniophora* and *Parasarocladium* species) and subsequently sent to the Genomics Unit at IPBLN-CSIC (Granada, Spain) for Illumina MiSeq PE300 v3 sequencing. The resulting raw sequencing data are available at the European Nucleotide Archive (ENA), EMBL-EBI, under accession no. PRJEB86993 (https://www.ebi.ac.uk/ena/browser/view/PRJEB86993).

### Bioinformatics Pipeline

The bioinformatics pipeline, from raw sequences to the operational taxonomic units (OTU) matrix, consisted of eight steps: (i) trimming of primers and removal of reads shorter than 200 pb using CUTADAPT [28]; (ii) quality filtering, dereplication, denoising, merging in contigs, removal of chimeras and creating the amplicon sequence variants (ASV) table, all of them by using DADA2 [29]; (iii) extraction of the 5.8S rRNA gene and ITS2 region with ITSx; (iv – for ITS2) clustering of ASVs into OTUs at 97% similarity and removal of singletons using VSEARCH [30]; (v – for ITS2) curation of OTU table with LULU [31]; (vi – for 5.8S) dereplication and sorting using VSEARCH; (vii – for both regions separately) taxonomic assignments of OTUs/ASVs against the UNITE database v. 9.0—UNITE general FASTA release for eukaryotes [32] using the basic local alignment search tool (BLAST) and the lowest common ancestor (LCA) analysis and finally (viii) merging the two taxonomic assignments to generate the final taxonomy using R.

### Data Filtering and Assessment of Controls and Replicates

The 3320 resulting OTUs were mostly assigned to the kingdoms *Fungi* (82.2%), *Viridiplantae* (5.9%) and *Alveolata* (2.7%), among others, with 6.9% of OTUs unidentified. The assessment of controls and technical replicates was performed on the fungal OTU matrix (2728 OTUs from 190 samples), following the code available at the Zenodo dataset (10.5281/zenodo.15488704). All mock samples showed identical patterns composed of the same four OTUs, which corresponded to the mock-community members, identified as *Papiliotrema terrestris*, *Cercospora sojina*, *Peniophora* sp. and *Parasarocladium wereldwijsianum*. They were also detected in some (4–15) environmental samples from hospitals, which is not surprising since mock-community members were isolated from air and dust samples from VR hospital. Number, identity and abundance of OTUs in the negative controls (negative samples, extraction negatives and PCR negatives) were checked and corrected considering their distribution in the environmental samples. A total of 21 OTUs were identified as potential contaminants, with high abundance in some negative controls and limited distribution in environmental samples, and consequently deleted from the matrix. The similarity of the community profiles for the 9 duplicated samples was confirmed by non-metric multidimensional scaling (NMDS) ordination, confirming the reproducibility of the DNA metabarcoding workflow. After removal of control samples and those replicates with lower numbers of OTUs and reads, the quality-filtered matrix contained 2688 OTUs from 167 samples.

### Airborne Fungal Load by qPCR

To complement the DNA metabarcoding data and allow the methodological comparison, airborne fungal load was quantified by qPCR using the universal fungal primers NL1f and LS2r targeting the 28S rRNA gene [33]. A standard curve was constructed using six serial decimal dilutions in triplicate of an equimolar mixture of fungal DNA extracts (*Trichoderma* and *Alternaria* species isolated from the hospital environment), ranging from 2 ng to 0.02 pg. qPCR assays were performed using the Sso advanced SYBR Green Supermix (BioRad, Hercules, CA, USA) as previously described by Martin-Sanchez et al. [34], including two reactions for each of the 72 air samples, two positive standards in duplicate and two negative controls without DNA. The 10-µl qPCR reactions were run in a CFX Connect Real-Time PCR Detection System (BioRad) with the following cycling parameters: 95 °C for 5 min followed by 40 cycles consisting of 5 s at 95 °C and 1 min at 60 °C and subsequent reading of fluorescence at 520 nm. To construct the melting curve, 60 steps of 5 s at increasing temperature by 0.5 °C (from 65 to 95 °C) and reading the fluorescence at 520 nm were added. Data analyses were conducted using the CFX Maestro software (BioRad). The resulting fungal loads were expressed as the concentration of fungal DNA in air (pg/m^3^).

### Statistical Analyses

Statistical analyses were conducted in R v 4.3.0 through RStudio v 2023.03.0 [35]. Tidyverse v 1.2.1 [36] and vegan v 2.6–4 [37] R packages were used for data manipulation, plotting and ecological analyses, respectively. The quality-filtered fungal OTU table was rarefied to the minimum number of reads per sample (13,257 reads) using the function *rrarefy* from vegan, which kept all samples (*n* = 167) but slightly decreased the number of OTUs (2652; 36 less). This rarefaction process randomly selects the same number of reads for all samples, correcting their differences in sample size for a proper assessment of both alpha and beta diversity. Different datasets were established for data comparisons: (i) all samples, (ii) all samples excluding soil samples and 9 extra samples (details in the “Sampling” section), (iii) winter samples, (iv) autumn samples and (v) air samples.

The normal distribution of alpha diversity data, fungal richness (observed number of OTUs) and Shannon index per sample was initially evaluated by the Shapiro–Wilk test. Considering that no data followed a normal distribution, variances of data for different sampling campaigns, hospitals, zones and sample types were evaluated by the non-parametric Kruskal–Wallis test and the post hoc Mann–Whitney-Wilcoxon test. Beta diversity patterns were assessed through NMDS ordination of environmental samples applying the function *metaMDS* from vegan, Bray–Curtis dissimilarity index and 200 random starts in search of a stable solution on the corresponding rarefied OTU tables. To evaluate the influence of sampling-related factors (sampling campaign, hospital, zone and sample type), environmental variables (particle counts, AT, RH, etc.) and airborne fungal load data (colony forming units—CFU and fungal DNA) on the fungal community composition, permutational multivariate analysis of variance (PERMANOVA; 999 permutations) was performed individually on each variable using *adonis2* from vegan. In addition, multivariate homogeneity of groups’ dispersions was tested by the vegan function *betadisper.* For further comparison between hospitals and zones, we calculated their total numbers of OTUs and overlaps. Fungal taxonomic composition, by sampling campaign, hospital, zone and sample type, was analyzed at the phylum, order and genus level due to the well-known limitations of species identification based on such short DNA fragments. To reveal significant associations (*p* < 0.05) between OTUs and sample type and hospital zone, an indicator species analysis was performed using *multipatt* from the Indicspecies R package v. 1.7.14 [38] on autumn and air datasets, respectively. To compare different methodological approaches, Spearman’s correlation coefficients were calculated between data from particle counts, culture-based variables (fungal CFU and number of fungal morphotypes) and DNA-based variables (fungal DNA and fungal richness). Complete codes for statistical analyses are available at the Zenodo dataset (10.5281/zenodo.15488704).

## Results

### DNA Metabarcoding Data Features and Alpha Diversity

High-throughput sequencing data were achieved for all air, dust and soil samples, while a considerable number of HTS (40 of 58; 68.9%) and vent (25 of 58; 43.1%) samples failed in the PCR rounds, especially for winter samples. Some sponge-stick samples got positive PCR results using 5-times diluted DNA extracts as templates, which indicated a probable release of PCR inhibitors from these sponges. This limitation was especially critical for studying surfaces with very low biomass such as HTS collected after cleaning from IB and IC zones, whose DNA yields are too low to allow further dilution.

**Fig. 1:**
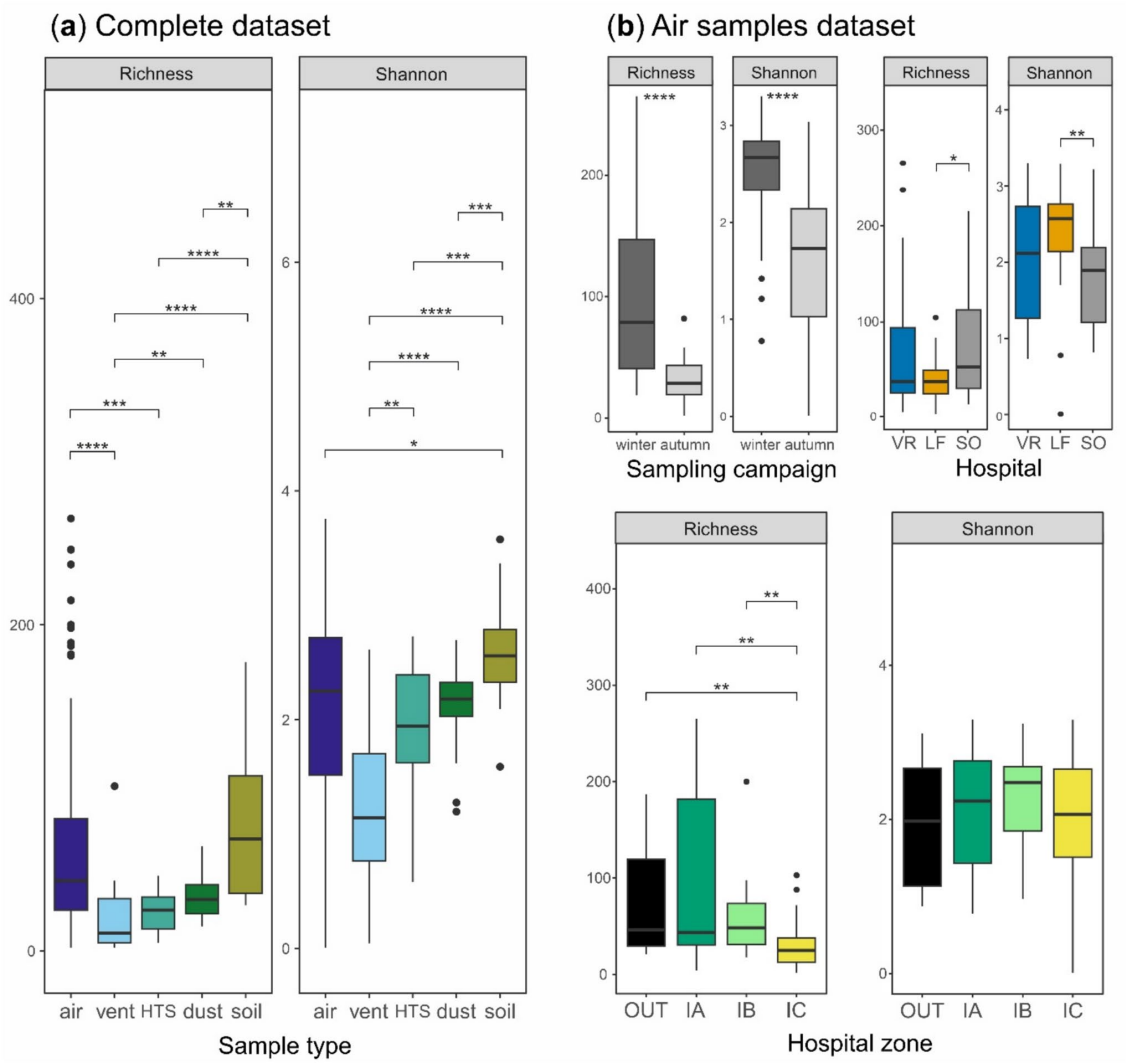
Alpha diversity measures, operational taxonomic unit (OTU) richness and Shannon index, for the hospital mycobiomes. **a** Comparison between sample types based on the complete rarefied matrix including 2652 OTUs from 167 samples. **b** Comparisons of other factors (sampling campaign, hospital and hospital zones) based on the air samples dataset (*n* = 72). Asterisks indicate significant differences according to the Mann–Whitney-Wilcoxon test (*p ≤ 0.05; **p ≤ 0.01; ***p ≤ 0.001; ****p ≤ 0.0001). Lower and upper box boundaries are the 25th and 75th percentiles, respectively; the line inside the box is the median; lower and upper lines are whiskers to minimum and maximum values, respectively

The number of reads per sample ranged from 13,258 to 154,726 (median = 40,232), and the number of OTUs per sample (fungal richness) ranged from 2 to 273 (median = 32). After rarefaction (to 13,258 reads per sample) of the data matrix, both richness and Shannon index data showed significant differences between the compared sample types (Fig. 1a). Soil and air samples achieved the highest fungal richness with median values of 68.5 and 43, respectively, which were significantly (*p* ≤ 0.001) different from those from indoor surface samples, vent (11) and HTS (25). Diversity of soilborne fungi was significantly (*p* ≤ 0.05) higher compared to the rest of the sample types including air samples.

To evaluate the influence of sampling-related factors (i.e. sampling campaign, hospital and zone) on alpha diversity, we focused on the air samples dataset (Fig. 1b) that is homogeneous and free of the bias mentioned for surface (sponge) samples. Interestingly, both fungal richness and Shannon index were significantly (*p* ≤ 0.0001) higher in air samples collected in winter compared to those taken in autumn. Richness of airborne fungi was slightly higher in SO hospital (median = 51.5) compared to VR (median = 36; non-significant difference, *p* > 0.05) and LF (median = 36; *p* ≤ 0.05). In contrast, fungal diversity (based on Shannon index) was significantly (*p* ≤ 0.01) higher in LF (median = 2.57) compared to SO (median = 1.89). When it comes to the comparison of hospital zones, the only significant (*p* ≤ 0.01) difference was the lower richness observed in intensive care units (IC; median = 23) compared to the rest of the zones indoors (IA and IB) or outdoors (OUT), whose median values ranged from 44 to 48.

Comparing with previous culture-dependent results for airborne fungi in winter [25], DNA metabarcoding achieved much higher fungal richness (19–265 OTUs per sample, median = 76; Fig. 1b) than the counts of cultivated morphotypes, which ranged 0–13 (median = 4.5) or 1–16 (median = 5) for DRBC and SDCA media, respectively. Therefore, the eDNA method on average increased 90.6–93.6% the estimates of fungal richness compared to morphotype counting on SDCA and DRBC, respectively.

### Fungal Community Composition

Based on a first exploration of beta diversity in the complete dataset (Supplementary Fig. S1), fungal community composition of the soil samples from hospital gardens showed clear differences from the rest of sample types (air and surface samples). Therefore, in order to better characterize the hospital mycobiomes, soil samples were excluded from the further analyses.

**Fig. 2:**
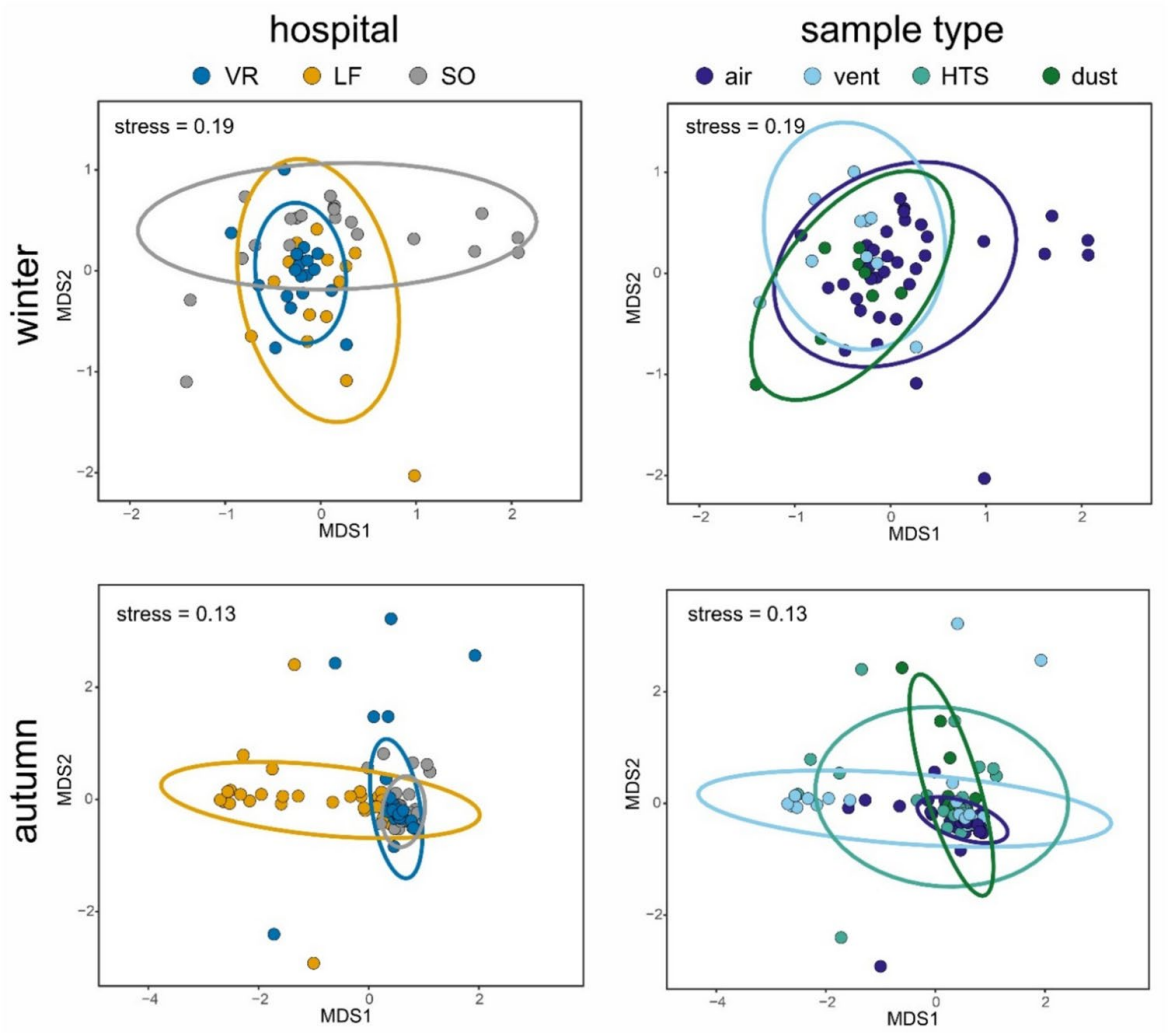
Non-metric multidimensional scaling (NMDS) ordination plots showing compositional variation of hospital mycobiomes sampled in winter (top; 1502 operational taxonomic units from 55 samples) and autumn (bottom; 822 OTUs from 85 samples). Points represent environmental samples, and their colors indicate hospitals (left) or sample types (right). Note: there are no HTS samples in the winter dataset as they failed in the PCRs

Beta diversity of the rest of the samples, collected in the two sampling campaigns (winter and autumn), is illustrated by hospital and sample type in Fig. 2, showing characteristic patterns for SO samples collected in winter and LF samples taken in autumn. Besides, according to PERMANOVA results (Table 1), the most important and significant (*p* < 0.05) explanatory factors for the observed variance of mycobiomes were the hospital where samples were collected, with R2 values ranged from 10.5 to 16.9% depending on the dataset, sample type (6.5–12.1%), zone (4.3–7.6%), sampling campaign (2.4–8.6%), airborne particle counts (2.8–8.6%) and environmental variables such as RH (1.9–6.4%), DP (1.8–5.8%) and WB (1.6–4.7%). Focusing on air samples, the two sampling campaigns showed distinct patterns with a higher beta dispersion in winter compared to autumn, which lack statistical significance (Fig. S2; *p* = 0.5). In addition to the mentioned explanatory factors, some culture-dependent variables (CFUs on SDCA and DRBC media) showed significant associations (3.1 and 4%, respectively) with the structure of airborne fungal communities (Table 1, Fig. S2).

**Table 1:** Permutational multivariate analysis of variance (PERMANOVA) results showing the significant explanatory variables for the observed variance in the complete, winter, autumn and air datasets (R2 values with *p* < 0.05), which contain 140, 55, 85 and 72 environmental samples, respectively, excluding soil samples

	R2 values (%) with *p* < 0.05 ^a^			
Variables	Complete (*n* = 140)	Winter (*n* = 55)	Autumn (*n* = 85)	Air (*n* = 72)
sampling_campaign	2.4	na	na	8.6
hospital	10.5	12.3	16.9	12.8
zone	4.3	7.6	7.2	6.7
sample_type	6.5	6.6	12.1	na
particles_0.3	4.8	4.3	6.9	6.5
particles_0.5	5.1	4.3	7.5	7
particles_1.0	4.9	4.4	7.4	6.8
particles_2.5	5.1	3.7	8.6	6.3
particles_5.0	3.5	ns	8.3	3.9
particles_10	2.8	ns	7.2	ns
RH	1.9	ns	ns	6.4
DP	1.9	5.8	ns	5.1
WB	1.6	4.7	ns	3.6
CFU_SDCA_air	na	na	na	3.1
CFU_DRBC_air	na	na	na	4

^a^Some variables, air temperature (AT) and qPCR-based fungal DNA concentration in air (fungal_DNA_air), were excluded because their R2 values were not significant (*p* > 0.05) for any of the datasets

*na* not applicable, *ns* not significant (*p* > 0.05), *RH* relative humidity, *DP* dew point temperature, *WB* wet-bulb temperature

After excluding soil samples, hospital mycobiomes were represented by 1900 OTUs mostly affiliated to the phyla *Ascomycota* (53% of OTUs), *Basidiomycota* (41.3%), *Chytridiomycota* (0.7%) and *Mucoromycota* (0.4%), with 4% of OTUs unidentified including those assigned as “*Fungi*_phy_*Incertae_sedis*” (i.e. uncertain fungal phylum). At lower taxonomical levels, hospital mycobiomes included members of 35 classes, 114 orders, 305 families, 643 genera and 535 species, after excluding the non-informative results at each taxonomic level (e.g. “*Incertea_sedis*” or “sp” items) (Supplementary Table S1).

Focusing on the indoor OTUs (1557 of 1900 OTUs – 81.9%), the overlap between the three hospitals was relatively low (9.25% of OTUs), with 73.5% of OTUs only found in one of the hospitals (33.2% in VR, 32.3% in SO and 8% in LF) (Fig. S3). 11.2% of OTUs were detected in all three indoor zones, while the proportions of OTUs detected in a single zone decreased from the most exposed areas (IA, entrance halls and waiting rooms; 41.3%) to the most protected areas (IC, intensive care units protected with HEPA filters; 9.9%). Following the same trend, the IA zone shared more OTUs with the IB zone (regular patient rooms) than with the IC zone (Fig. S3).

According to the mean relative abundances per sample, the top taxa were the phyla *Ascomycota* (85.4–95%, ranged by sample type from air to dust) and *Basidiomycota* (4.5–14.3%, dust–air) (Fig. S4); the orders *Capnodiales* (36–60.9%, HTS–vent), *Pleosporales* (15–23.5%, air–dust), *Dothideales* (2.5–8.4%, vent–dust), *Saccharomycetales* (0.03–20%, dust–HTS) and *Eurotiales* (0.2–8.5%, dust–HTS) (Fig. S5); and the genera *Cladosporium* (36–59.3%, HTS–vent), *Alternaria* (5.4–9.4%, vent–HTS), *Aureobasidium* (2.3–8.4%, vent–dust), *Candida* (0.001–15%, dust–HTS), *Penicillium* (0.1–6.1%, dust–HTS) and *Neodidymelliopsis* (1.6–5.4%, air–vent) (Fig. S6).

**Fig. 3:**
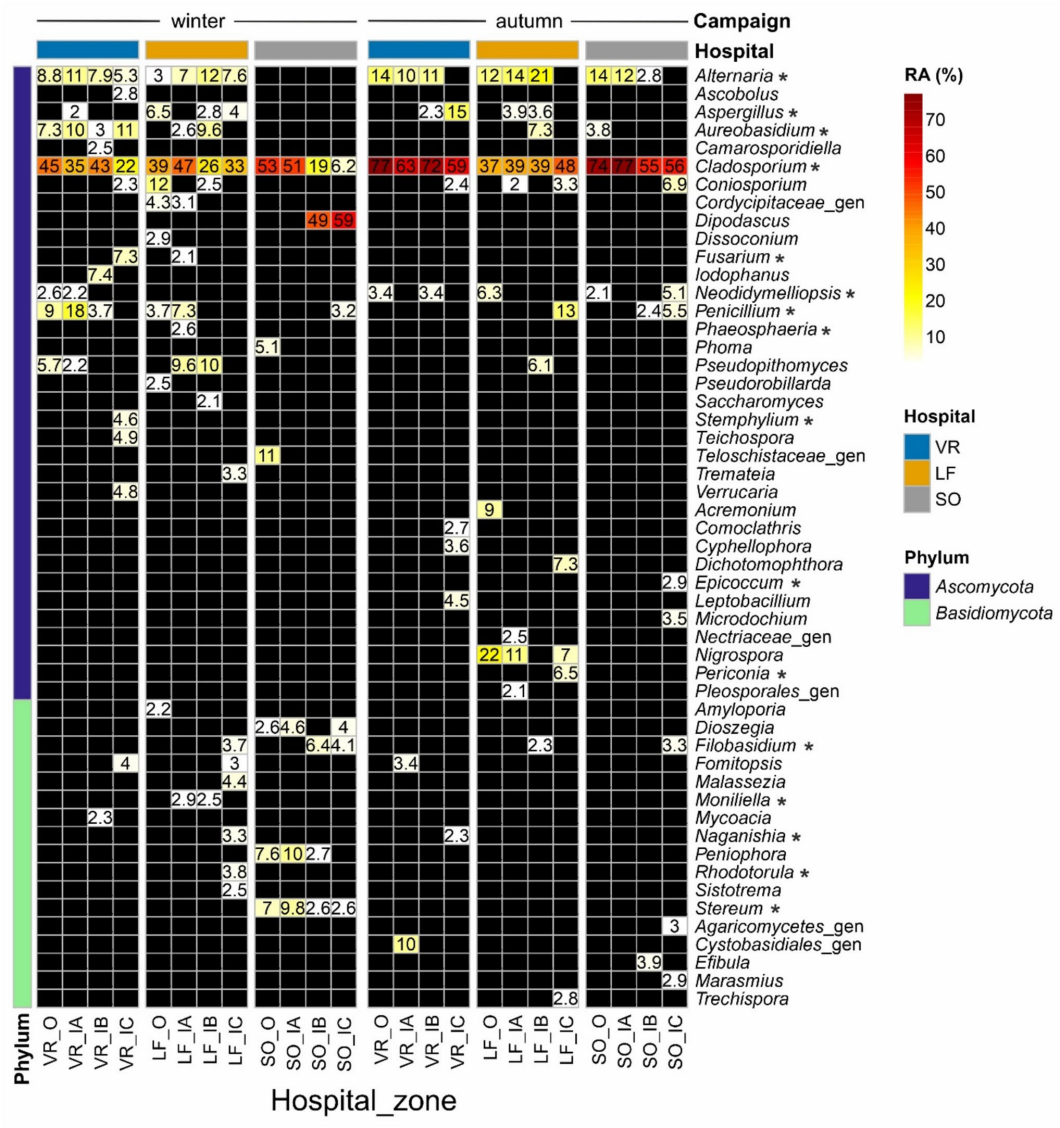
Heatmap showing the most abundant genera (> 2% relative abundance of reads—RA) identified in air samples by sampling campaign (winter-left vs. autumn-right), hospital and zone. Asterisks indicate genera detected in air samples collected in winter using culture-dependent methods [25]. Note that some groups were identified as uncertain genera (“_gen”) assigned to higher taxonomical levels (family, order or class), and black cells do not necessarily indicate absence but RA < 2%

**Fig. 4:**
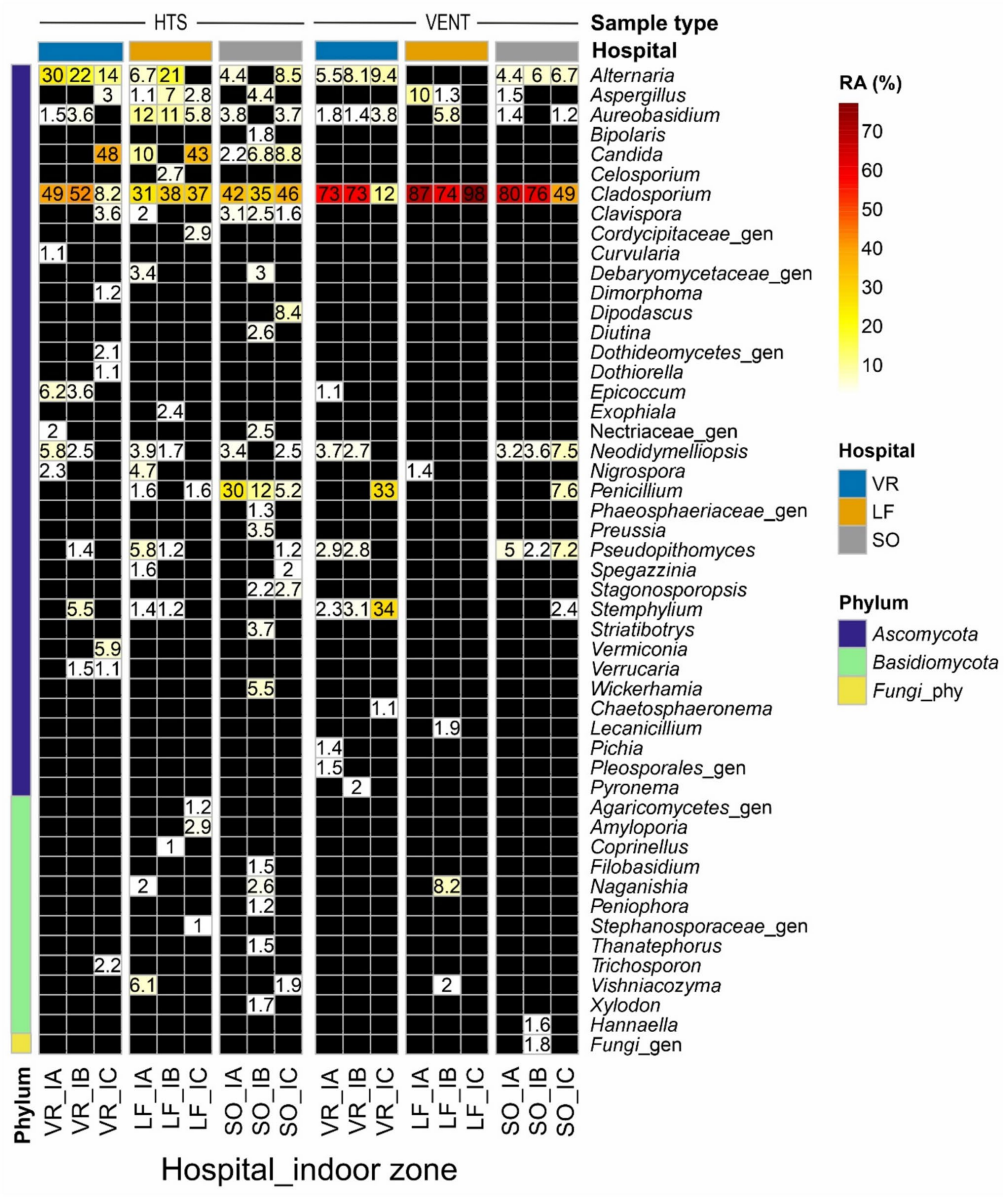
Heatmap showing the most abundant genera (> 1% relative abundance—RA) identified in surface (HTS and vent) samples collected in autumn by hospital and zone. Note that some groups were identified as uncertain genera (“_gen”) assigned to higher taxonomical levels (family, order or class), and black cells do not necessarily indicate absence but RA < 1%

*Cladosporium* clearly was the dominant (top 1) genus in hospital mycobiomes, widely distributed in all sampling campaigns, hospitals, zones and sample types, with relative abundances by zones ranged from 6.2 to 99% in air (Fig. 3), and from 8.2 to 98% in surfaces (Fig. 4, autumn campaign). Although this genus showed slightly higher abundances in air and vent samples, as well as OUT and IA zones, it also colonized efficiently clean HTS surfaces and clean or protected zones (IB and IC). Similarly, but at lesser extent, *Alternaria*, *Aureobasidium*, *Penicillium*, *Neodidymelliopsis*, *Aspergillus*, *Pseudopithomyces* and *Stemphylium* were widely distributed in both air and surfaces from the three hospitals. In particular, *Aspergillus* showed airborne relative abundances by zones ranged from 0.02 to 15% of reads, only absent in the air samples collected in autumn from OUT, IA and IC zones at SO hospital. The opportunistic pathogen *Aspergillus fumigatus* was very scarce in our eDNA dataset, only accounting for a few rarefied reads (≤ 70) in three air samples from SO and LF, and one HTS sample from VR. The basidiomycetous yeasts *Naganishia* and *Vishniacozyma* also showed a wide distribution, detected in 46.4% (65) and 44.3% (62) of samples, respectively, from all hospitals, zones and sample types; with relative abundances ≤ 16.3% and ≤ 8.7% of rarefied reads per sample, respectively.

The aerial distribution in winter of the genera *Dipodascus*, *Dioszegia*, *Peniophora* and *Stereum* was mostly associated with the SO hospital. In particular, the genus *Dipodascus* was detected in all SO air samples collected in winter, increasing from outside (0.6%), IA (1.5%), IB (49%) to IC (59%); and detected in HTS samples from IB (0.09%) and IC (8.4%) zones in autumn (Figs. 3 and 4). Remarkably, the genera *Candida* and *Filobasidium* were proportionally more abundant in clean patient rooms (IB zone) and HEPA-protected intensive care units (IC) (Figs. 3 and 4). Besides, one OTU affiliated to the genus *Leptobacillium* (*L. chinense*) was identified as an indicator (IndVal = 68.2%, *p* < 0.05) for the zone IC. This was detected in nine air samples from the intensive care units of the three hospitals (VR, LF and SO in winter, and VR and LF in autumn), with a mean relative abundance of 2.8 ± 1.1%.

Some yeast genera, such as *Candida* and *Clavispora*, showed higher relative abundance in HTS samples (Fig. 4), and some of their species were identified as significant indicators for this type of surfaces (Table 2; IndVal > 54.9%, *p* < 0.05). In particular, two indicator species were identified as *Candida parapsilosis* and *Candida dubliniensis*, where the latter one was also identical to *Candida albicans* (100% similarity in ITS2 sequence). *C. parapsilosis* was prevalent in our survey, present in all sampling campaigns, hospitals and zones, mostly in HTS but also in air and vent samples. In addition, the indicator species analysis found some members of the orders *Pleosporales* (genera *Epicoccum* and *Dimorphoma*), *Chaetothyriales* (*Lithohypha*) and *Mycosphaerellales* significantly associated with the outdoor dust samples (IndVal > 54%, *p* < 0.05; Table 2).

**Table 2:** OTUs identified as indicator fungal species (Indicator values—IndVal > 50%, *p* < 0.05) for the different sample types using the R package *indicspecies* [38] on the autumn dataset (822 OTUs from 85 samples)

OTU id	Phylum	Order	Genus/species	RA (%)	# of samples	IndVal (%)
HTS indicators						
LF2-A-IA1_Seq14	*Ascomycota*	*Saccharomycetales*	*Candida parapsilosis*	1.5	23	78.1
LF2-S-IC2_Seq70 ^a^	*Ascomycota*	*Saccharomycetales*	*Candida dubliniensis*^a^	1.6	9	55.8
LF2-A-O3_Seq94	*Ascomycota*	*Saccharomycetales*	*Clavispora* sp.	0.3	13	54.9
Air indicators						
LF2-A-O1_Seq815	*Basidiomycota*	*Agaricales*	*Agaricus bitorquis*	0.03	12	53.4
Outdoor dust indicators						
LF2-A-O1_Seq192	*Ascomycota*	*Pleosporales*	*Epicoccum draconis*	0.6	25	83.2
LF2-D-O2_Seq39	*Ascomycota*	*Chaetothyriales*	*Lithohypha aloicola*	0.4	12	79.3
LF2-D-O1_Seq84	*Ascomycota*	*Pleosporales*	*Dimorphoma saxea*	0.1	8	76.3
LF2-A-IB1_Seq164	*Ascomycota*			0.07	9	71.5
LF2-So-O1_Seq51	*Ascomycota*	*Pleosporales*		0.3	4	66.6
LF-A-IC3_Seq95	*Ascomycota*	*Mycosphaerellales*		0.3	6	54. 8

^a^OTU representative sequence showing 100% similarity with sequences from two *Candida* species: *C. dubliniensis* and *C. albicans*

### Fungal Load by qPCR and its Correlation with Different Approaches

Airborne fungal loads assessed by qPCR (fungal DNA) showed similar patterns to those previously reported for CFU and particle counts [25]: (i) overall decreasing indoor gradient from the most exposed and occupied zones (IA) to the most protected zones (IC), (ii) higher fungal load in SO hospital in winter compared zone-by-zone to VR and LF hospitals and (iii) much higher outdoor fungal loads in autumn compared to winter for the three hospitals (Fig. S7). It is worthy to mention that, in winter, fungal loads at entrance halls and waiting rooms (IA zones) were higher than those measured outdoors.

**Fig. 5:**
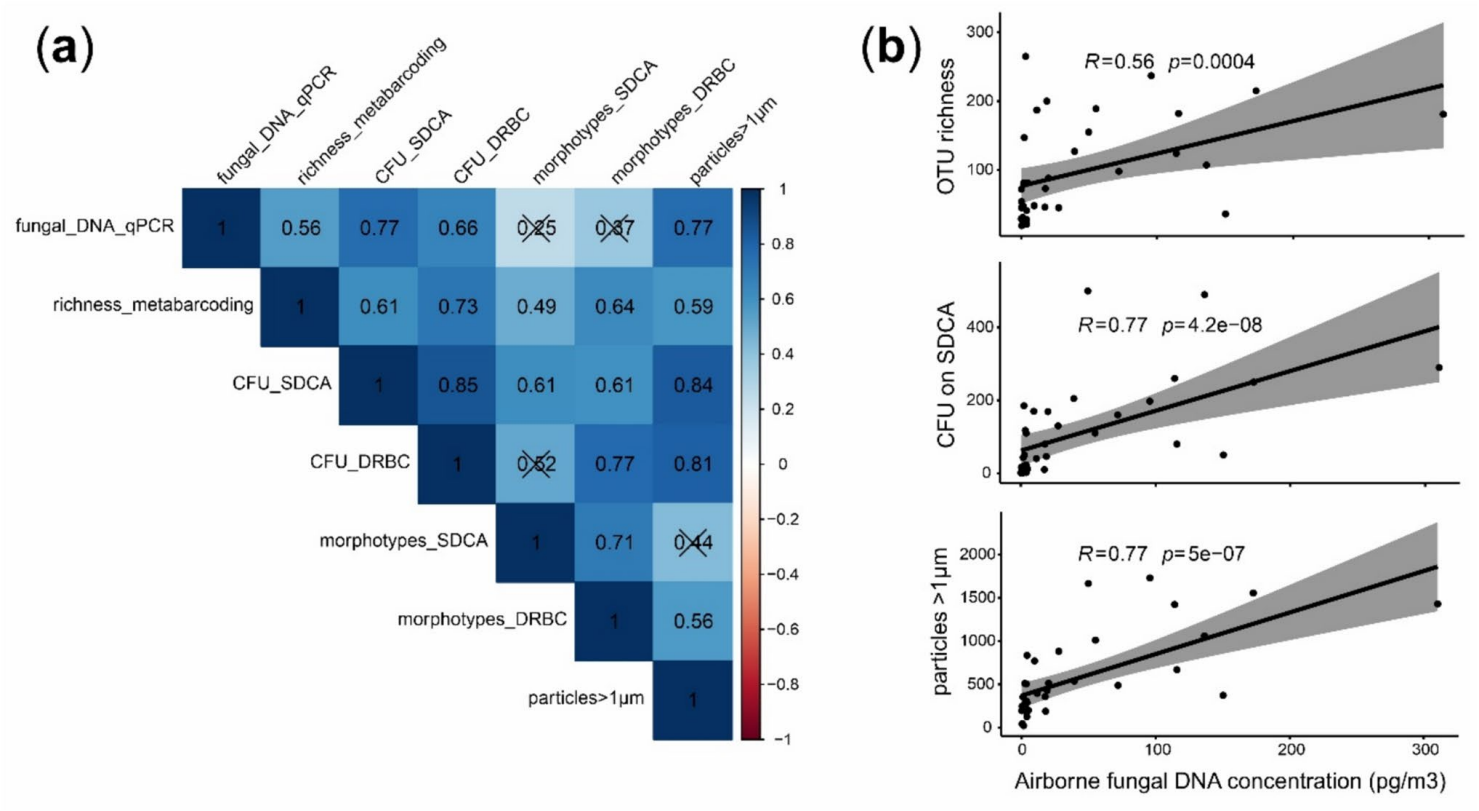
**a** Spearman’s correlation matrix between variables from different approaches (environmental DNA, culturing and particles), some related to fungal load such as fungal DNA by qPCR (fungal_DNA_qPCR), colony forming units on Sabouraud dextrose chloramphenicol agar (CFU_SDCA) and dichloran rose bengal chloramphenicol agar (CFU_DRBC) media, and particles with > 1 µm diameter (particles_1); and those related to fungal diversity such as observed richness (richness_metabarcoding) and observed colony morphotypes (morphotypes_SDCA and morphotypes_DRBC). **b** Details of correlations between qPCR-based fungal DNA, OTU richness, CFU on SDCA and particles with > 1 µm diameter. Dataset of air samples collected in winter (*n* = 36). Non-significant correlation coefficients (*p* > 0.05) are shown crossed out

The three methodological approaches used to estimate/quantify fungal loads (qPCR, CFUs and particle counts) showed a good agreement between them according to the high and significant positive Spearman’s correlation coefficients (0.66–0.77; *p* < 0.05) (Fig. 5). Besides, qPCR-based fungal DNA data were significantly correlated with the OTU richness (0.56; *p* < 0.05), but not with the number of observed morphotypes on culture media (0.25–0.37; *p* > 0.05) (Fig. 5).

## Discussion

### Alpha Diversity Patterns

The moderate ranges of fungal richness found in the studied hospitals, with air samples ranged from 2 to 277 OTUs (Fig. 1a), were similar to previous DNA metabarcoding studies in hospitals: Habibi et al. [22] detected a slightly lower number of OTUs (19–33) in air samples from hospitals in Kuwait, while Núñez and García [23] and Chen et al. [24] found higher numbers of ASVs (100–800) in air samples from hospitals in Spain and China, respectively. This richness increase in the latter studies may be partially due to the different bioinformatics approaches analyzing directly ASVs, compared to our more stringent pipeline that includes further clustering in OTUs and the subsequent correction of over-splitting of OTUs by LULU.

Fungal richness in indoor surfaces (vent and HTS) was significantly (*p* ≤ 0.001) lower than in air samples, which is most likely due to the low microbial load in many of these surfaces, especially those from IB (regular patient rooms) and IC (intensive care units) zones that were cleaned before sampling. To the best of our knowledge, it is the first time that hospital surface-colonizing fungi have been characterized by DNA metabarcoding, which hinders the discussion of these findings. Furthermore, the significantly (*p* ≤ 0.01) lower richness in ICUs compared to the rest of the hospital zones was a foreseeable result considering the higher access restrictions and ventilation control through HEPA filters in this critical zone.

Although our sampling scheme was not aimed to evaluate seasonal changes in the hospital mycobiomes, overall, significantly higher fungal richness was observed in the samples collected in winter (January–February) compared to autumn (September–October). In contrast, fungal load (both CFU and DNA measured by qPCR) and particle data showed the opposite trend in the three studied hospitals, with much higher levels in autumn [25] (Fig. S7). Most likely, the increased concentrations in autumn of some dominant outdoor airborne fungi, such as *Cladosporium*, which represented 77, 37 and 74% of reads outside VR, LF and SO hospitals, respectively (Fig. 3), and *Alternaria* (14, 12 and 14%, respectively), have caused decreased OTU richness hiding some less abundant OTUs by the well-known PCR biases. They can be especially important when analyzing the typical low-biomass samples (air, dust and surfaces) from the built environment as pointed out in previous studies [39–41]. In this regard, the dominance of these genera was also reflected in the indoor surfaces sampled in autumn, with *Cladosporium* reaching 73–87% in vent samples and 31–49% in HTS from IA zones, and *Alternaria* attaining 4.4–5.5% in vent and 4.4–30% in HTS at IA zones (Fig. 4). Adams et al. [40] reported that indoor residential surfaces mainly act as passive collectors of airborne fungi, which in turn are mostly coming from outdoors.

Previous eDNA studies analyzing dust samples from residential buildings in California (USA) and Munich (Germany) reported higher fungal richness in winter than in summer [41, 42]. On the contrary, Estensmo et al. [43] found the highest fungal richness in dust samples collected in late summer and autumn from daycare centres in Oslo (Norway). Núñez and García [23] did not find relevant differences in fungal richness between the air samples collected in winter and summer from a hospital in Madrid (Spain). However, the latter study only considered two sampling points, one indoors and one outdoors.

### Driving Factors for Hospital Mycobiomes

A considerable fraction of the variation in community composition could be explained by several factors related to climate (sampling campaigns in winter or autumn) and the geographical location of the three studied hospitals, all of them in highly urbanized areas of separate (300–700 km distance) cities with different climatic features: inland Mediterranean climate in Seville (VR), continental climate in Leganes (SO, Madrid) and coast Mediterranean climate in Valencia (LF). Previous studies have demonstrated that indoor fungal communities are influenced by multiple factors, mainly climate-/geography- (through outdoor air), building- and occupant-related variables [21, 44, 45], as well as fungal spore dispersal and colonization [46, 47].

Besides, the hospital zone where the sample was collected (OUT, IA, IB or IC) also had a significant influence on its fungal community composition, which is probably due to the different protection degree of zones against the influx of aerosols coming from both outdoor air and occupants. As proved by culturing and particle counting [25], our study zones showed significantly different concentrations of aerosols including airborne fungi. Thus, it is not surprising that they also differ in fungal community composition. Other authors have also reported changes in fungal load and composition depending on the sampled sites inside hospitals [48, 49]. However, this is the first eDNA-based study reporting on the main determinants for hospital mycobiome, comparing different zones and substrates (sample types).

Some IAQ variables measured at the specific sampling point and time, such as RH, DP, WB and particle counts, showed relatively low but significant explanatory power. Most likely, the association of RH, DP and WB is simply reflecting the differences between the two sampling campaigns/seasons (winter and autumn), characterized by different humidity conditions. In studies with higher numbers of buildings at a relatively large geographical scale, it is recommendable to assess the effect of more representative climatic variables, such as mean temperature, precipitation or relative humidity in a certain time period before sampling. However, we discarded these analyses since our study was focused on three hospitals from different cities and climatic areas; thus, any climatic variable will covary with the “hospital” factor. When it comes to the particles, they were positively correlated to fungal load (both CFU and DNA assessed by qPCR) and fungal richness (Fig. 5); thus, particulate matter should also be closely related to community composition. Previous studies have reported that the fungal taxonomic composition of bioaerosols varies strongly by their aerodynamic diameters and season [44, 50–52], with allergenic fungi (e.g. *Alternaria alternata* and *Epicoccum nigrum*) mostly clustered in the highest fraction (> 9 µm), and some opportunistic human pathogens (e.g. *Aspergillus fumigatus* and *Schizophyllum commune*) grouped in lower size ranges, typically < 4.7 µm diameter [51].

### Taxonomic Composition

In general, the taxonomic composition of the hospital mycobiomes revealed by DNA metabarcoding was similar to that recently reported by culture-based approaches [25]. Both methods showed the same dominant and prevalent genera in air and surfaces from all hospital zones: *Cladosporium*, *Alternaria*, *Aureobasidium*, *Penicillium* and *Aspergillus*. All of them have been widely reported in the built environment including healthcare settings [17, 52, 53]. According to the eDNA data, the genera *Neodidymelliopsis* and *Stemphylium* were also widely distributed across the three hospitals, while only a few isolates of them could be cultivated from air samples collected at VR. Both genera belong to the *Pleosporales* order and include saprophytic and plant pathogenic species; thus, they have likely been transported into hospitals by the outdoor air. *Stemphylium* has been reported as an important allergenic genus associated with rhinitis and asthma cases [54]. Comparing the airborne genera detected in winter (all hospitals and zones together) by the two approaches, 75.4% (43 of 57) of cultivated genera were detected by DNA metabarcoding, while only 6.8% (43 of 624) of genera identified by eDNA could be isolated by culturing. The latter overlap increased to 34.8% (16 of 46) when focusing on the most abundant (> 2%) genera (Fig. 3).

The DNA metabarcoding approach has also revealed new findings that were previously overlooked by culture-based analyses: (i) the prevalence of *Pseudopithomyces* in all studied hospitals, zones and substrates; (ii) the dominance of *Dipodascus* in clean patient rooms and ICUs from the SO hospital, detected in both air and HTS samples; and (iii) the wide distribution of *Vishniacozyma* in all hospitals, zones and sample types. Andersen et al. [55] reported *Pseudopithomyces* species (*Ps. chartarum* and others) as the most prevalent moisture indicator fungi in dust samples from Danish homes, which were uniquely detected by DNA-based methods, like in our studies. Nevertheless, this genus was formerly named as *Pithomyces*, a common plant saprotroph that has occasionally been isolated from clinical specimens such as superficial tissues and respiratory tract samples [56], and reported as a causal agent (*Pithomyces chartarum*) of invasive pulmonary disease in a non-immunocompromised patient [57]. This species was also detected in a few air and vent samples from VR and SO hospitals by culturing [25]. The genus *Dipodascus* is a typical member of the human mycobiota, colonizing skin, oral cavity and respiratory tract, which has also been associated with invasive infections in immunosuppressed individuals [58]. Further studies should be conducted to monitor the distribution of this genus in the hospital environment and to assess its associated risk of nosocomial infections. *Vishniacozyma victoriae* (formerly *Cryptococcus victoriae*) has been reported as a prevalent species in indoor dust samples playing an important exposure role for allergic airway diseases, associated with both protective and inflammatory effects [59, 60].

Our preceding study thoroughly discussed the pathogenic potential of the fungi isolated from these three hospitals, identifying 36 species previously reported as opportunistic human pathogens, which belong to the genera *Alternaria*, *Aspergillus*, *Aureobasidium*, *Beauveria*, *Candida*, *Clavispora*, *Cystobasidium*, *Filobasidium*, *Fusarium*, *Hyphopichia*, *Lodderomyces*, *Meyerozyma*, *Moesziomyces*, *Mucor*, *Penicillium*,* Pichia*,* Pithomyces*,* Purpureocillium*, *Pseudozyma*, *Rhinocladiella*, *Rhizomucor*, *Rhizopus*, *Rhodotorula*, *Schizophylum*, *Scolecobasidium*, *Talaromyces* and *Trichosporon* [25]. The current eDNA survey detected 92.5% of them (25 of 27, except *Pseudozyma* and *Rhizomucor*) and likely many other potentially pathogenic taxa into the 535 species list (Table S1). However, considering the well-known limitation of DNA metabarcoding for providing reliable taxonomic assignments at the species level, based on short ITS sequences, we decided do not discuss further about the pathogenicity of the assigned species. Both studies ([25] and this one), as almost all indoor fungal assessments in hospitals, are lacking highly reliable identification at the species level, which may be only achieved applying additional time- and money-consuming analyses on cultivated strains, such as detailed morphological characterization of microscopic reproductive structures by expert taxonomists and/or multigene phylogenies.

When it comes to the fungal priority pathogens listed by WHO [5], our hospital survey detected *C. parapsilosis* (“high-priority” pathogen) widely distributed in both surface and air samples, likely *C. albicans* (“critical-priority”) in some HTS and air samples, very little *A. fumigatus* (“critical-priority”) in four samples and diverse OTUs affiliated with the “high-priority” taxa *Mucorales* and *Fusarium*. *C. parapsilosis* is widely distributed in nature, efficiently colonizes the human skin as a commensal and also causes IFI in immunocompromised individuals. In the last years, numerous *C. parapsilosis* outbreaks have been reported in hospitals around the world, with a continuous increase in the isolation of fluconazole- and voriconazole-resistant strains [61, 62]. The cross-transmission of *C. parapsilosis* has been suggested because of the genetic correlation observed between clinical and environmental strains [63]. We also isolated this species from HTS samples of the three hospitals using selective conditions, Brilliance Candida Agar incubated at 37 °C [25]. *A. fumigatus* is a common mold species isolated from (both outdoor and indoor) air samples, including those collected from hospitals [17, 53]. However, the prevalence and relative abundance of this opportunistic pathogen in our eDNA-based survey were much lower than in previous reports, typically based on culture-dependent methods. Likely, the standard cultivation protocols, using rich media incubated at high temperatures, can enhance the isolation of certain fungi such as *A. fumigatus* against other fungal groups, resulting in an overrepresentation of their relative abundances. In this regard, our previous study [25] reported a considerable increase in the isolation rate of *A. fumigatus* when the incubation temperature was increased from 28 to 37 °C, using the same medium (SDCA). It is also worthwhile to mention that no *Candida auris* sequence was detected in this study despite the long outbreak of this “critical-priority” pathogen in LF hospital [64]. This negative result is most likely due to the selection of clean rooms and ICUs, without previous *C. auris* report, in our sampling scheme.

Interestingly, some relevant trends found, such as the associations of *Candida* and *Clavispora* with HTS samples, *Stereum* and *Filobasidium* with the SO hospital and the wide distribution of *Naganishia*, were supported by both culture-dependent and eDNA data. *Candida* and *Clavispora* (including *Clavispora lusitaniae*, teleomorph of *Candida lusitaniae*) are well-known commensal fungi related to the human skin, which mostly spread through fomite surfaces and can cause IFI in immunocompromised patients [65–67]; thus, it is logical to find them enriched on HTS samples. The particularly high proportion of *Candida* in previously cleaned HTS from intensive care units (IC zones) highlights the high sensitivity of DNA metabarcoding, as well as the important health risk related to the presence of this opportunistic pathogen on surfaces that are in close contact with critical patients. Nevertheless, this methodology, lacking a viability assessment (e.g. RNA-based analysis or PMA treatment), does not distinguish whether the detected eDNA came from live cells/spores or dead cell debris. Likewise, the high relative abundances of *Filobasidium* and *Naganishia* in the air of patient rooms (IB zones) and intensive care units (IC) may be due to its association with the occupants’ skin [68, 69]. The previous culture-based study also reported a high proportion of basidiomycetous skin-associated yeasts, *Naganishia*, *Moliniella*, *Rhodotorula* and *Filobasidium*, in air samples from the IB zones [25]. Lax et al. [70] demonstrated that bacterial communities on patient room surfaces, particularly on bedrails, consistently resembled the skin microbiota of the patients occupying the rooms.

To the best of our knowledge, this is the first study linking the genus *Leptobacillium* to the air of intensive care units specifically. This belongs to the *Cordycipitaceae* family and has been reported as a common taxon in aerosols from hospitals and a subway station [22, 71], as well as an entomopathogenic forest fungus that parasitizes many insects including mites [72].

### Pros and Cons of eDNA Methods for Microbiological Assessment in Healthcare Settings

This comparative study demonstrates the higher sensitivity of DNA metabarcoding against culture-based approaches, which allowed us to drastically increase (90–94%) the number of observed species (fungal richness) from the hospital environment including numerous non-cultivable fungi. Previous studies have reported this key advantage of DNA metabarcoding to provide more comprehensive inventories of different organisms, such as plants, fish and microbes, in a variety of environments compared to traditional surveys [16, 73–75]. Besides, eDNA approaches are not dependent on cultivation or highly skilled taxonomists able to interpret the slight morphological differences between taxa. Therefore, they can become standard and reliable protocols for regular molecular biology laboratories with available PCR machines and (in-house or external) NGS services, as long as they are carefully designed for the specific goals and include the appropriate controls to overcome the eDNA shortcomings.

Regardless of the target organisms, all steps of DNA metabarcoding protocols, from sampling, DNA extraction, PCR, library preparation, NGS to bioinformatics and data analyses, can be affected by relevant biases [16]. In particular, for the environmental assessment in healthcare settings, DNA metabarcoding has three key limitations: (i) the lack of distinction between eDNA coming from living/viable/active organisms and those from dead debris, (ii) the expected low biomass/DNA content in the typical environmental samples from buildings (i.e. air, dust and surface) and (iii) the consequent high risk of cross contamination between samples. The latter two closely related points require several negative controls (negative samples such as unused swabs, sponges or air filters, and blanks at DNA extraction and PCR steps) in addition to positive/mock controls and technical replicates. Such controls make DNA metabarcoding assays more robust and reliable, but also more time-consuming and expensive, which hinder their implementation in the routine environmental monitoring programmes at healthcare buildings.

Preventive medicine departments at hospitals demand fast quantitative methods to assess the general microbial (fungal and bacterial) load in environmental samples, as well as the specific detection and quantification of some clinically relevant taxa associated with hospital outbreaks (e.g. *A. fumigatus*, *C. auris* and more recently *C. parapsilopsis*). This study has demonstrated that fungal load data from different approaches, CFUs, particle counts and qPCR, were highly and significantly correlated. Therefore, for both general and specific environmental assessments, culture-dependent approaches should be combined with these valuable complementary techniques (particle counting and qPCR), at least for those samples with known sampled amounts (volume or surface) that allow the quantification of the targets. The eDNA-based qPCR offers diverse possibilities from general bacterial and fungal protocols ([34] and this study) to more specific protocols, e.g. those targeted to *Aspergillus* sections [19]. In this regard, many other specific and highly sensitive qPCR protocols that have been initially developed for clinical diagnostics of fungal pathogens may be further explored for potential use on environmental samples from controlled hospital rooms.

## Conclusions

DNA metabarcoding, despite the discussed limitations, can be a useful approach to characterize in depth the microbial communities inhabiting the hospital environments, including fungi, bacteria and arthropods, as well as to reveal the most relevant drivers for the community composition. Such complete datasets can provide relevant information to assist hospital managers under certain demanding situations, for instance, as environmental assessments before opening new hospital units, during construction works or reported microbial outbreaks. eDNA assessments under normal operating hospital conditions, like in this study, contribute to the knowledge about the “normal baseline” indoor microorganisms (as defined by Andersen et al. [55]) in this kind of public buildings. This is crucial to identify community imbalances that might imply a health risk for exposed occupants, mostly patients but also healthcare workers, causing microbial outbreaks. Under such critical situations, it is important to implement infection control measures to minimize cross-transmission and the spread of the opportunistic pathogens involved.

In this particular study, our fungal eDNA approach was able to achieve the following specific conclusions: (i) DNA metabarcoding revealed a much more comprehensive inventory of hospital-inhabiting fungi, including those from air, vents and HTS; (ii) this methodology showed the significant influence of climate, geographical location, hospital zones and sample type on both alpha diversity and community composition of hospital mycobiomes; (iii) there was a high agreement between the most abundant and prevalent hospital fungi found by cultivation and DNA metabarcoding, which mainly belonged to the genera *Cladosporium*, *Alternaria*, *Aureobasidium*, *Penicillium* and *Aspergillus*; (iv) both approaches detected a variety of taxa that have previously been reported as opportunistic human pathogens, including some priority pathogens listed by WHO such as species of *Mucorales*, *Candida* and *Fusarium*; and (v) the fungi-specific qPCR protocol proved to be a fast and reliable method to quantify the fungal load in samples from the hospital environment; thus, this could be a good eDNA-based alternative/complement for CFU and particle data. In summary, this comparative study provides relevant insights to improve the environmental microbial assessments in healthcare settings, which are vital for guaranteeing a proper indoor air quality and preventing nosocomial infections.

## Supplementary Information

Below is the link to the electronic supplementary material.

ESM 1 Supplementary Material 1 (XLSX 283 KB)

ESM 2 Supplementary Material 2 (PDF 608 KB)

## Data Availability

The dataset supporting the conclusions of this article is available at Zenodo repository (10.5281/zenodo.15488704), which includes the initial complete OTU table, the final rarefied matrix for fungi, metadata including sampling, particle and culture-dependent data, taxonomic assignment of OTUs, detailed R scripts for reproducing data preparation and data analyses, including all figures and tables from this study. Raw sequencing data are available at the European Nucleotide Archive (ENA), EMBL-EBI, under the study accession no. PRJEB86993 (https://www.ebi.ac.uk/ena/browser/view/PRJEB86993).
