# Supplementary material for: Contribution of DNA Metabarcoding to the Environmental Fungal Assessments in Hospitals
Source: Microb Ecol. 2025 Nov 24;89(1):3. doi: 10.1007/s00248-025-02626-w (PMC12722384; doi:10.1007/s00248-025-02626-w)
Supplement: Supplementary file 2 — Supplementary Material 2 (PDF 608 KB) [file 248_2025_2626_MOESM2_ESM.pdf]

Supplementary Material

Journal name: *Microbial Ecology*

Article title: ***Contribution of DNA metabarcoding to the environmental fungal assessments in hospitals***

Authors: Laura García-Gutiérrez[a], Emilia Mellado[b,c] & Pedro M. Martin-Sanchez[a*]

[a]: Laboratorio de Microbiología Ambiental y Patrimonio Cultural, Instituto de Recursos Naturales y Agrobiología de Sevilla (IRNAS), Consejo Superior de Investigaciones Científicas (CSIC), Avda. Reina Mercedes 10, 41012 Seville, Spain. lauragg@irnas.csic.es; pmartin@irnase.csic.es

[b]: Laboratorio de Referencia e Investigación en Micología, Centro Nacional de Microbiología, Instituto de Salud Carlos III (ISCIII), Ctra. de Pozuelo 28, 28222 Majadahonda, Madrid, Spain. emellado@isciii.es

[c]: Centro de investigación Biomédica en Red – Enfermedades Infecciosas (CIBERINFEC-CB21/13/00105): ISCIII, Majadahonda, Madrid, Spain.

*Corresponding author: pmartin@irnase.csic.es

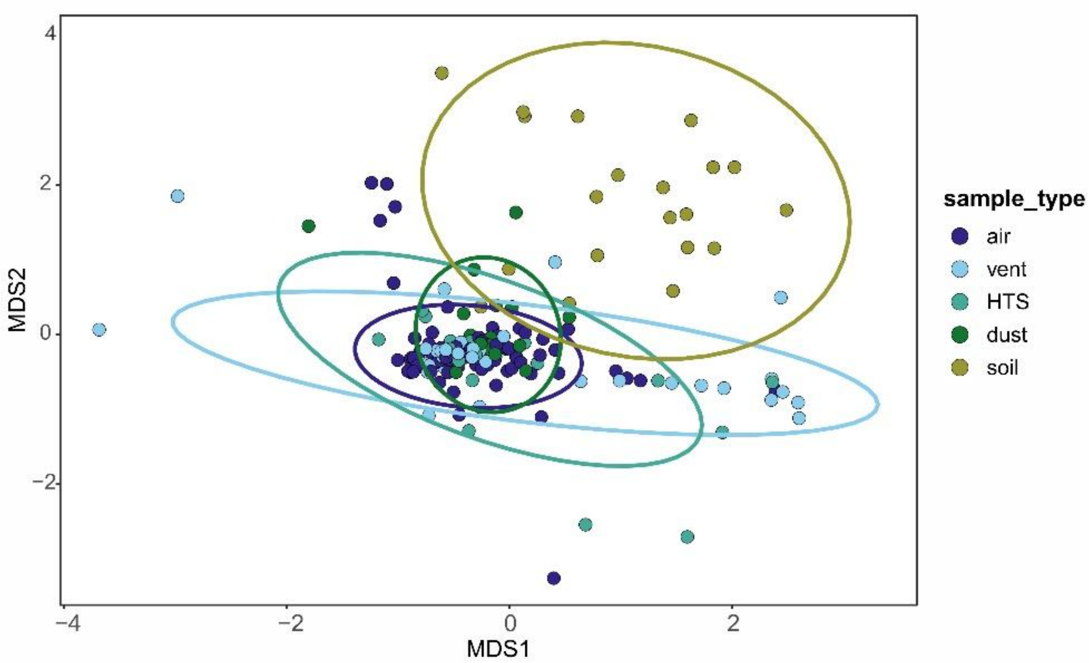

Figure S1. NMDS ordination plot showing compositional variation of the complete dataset including soil samples (2,652 OTUs from 167 samples). Points represent environmental samples, and their colors indicate sample types.

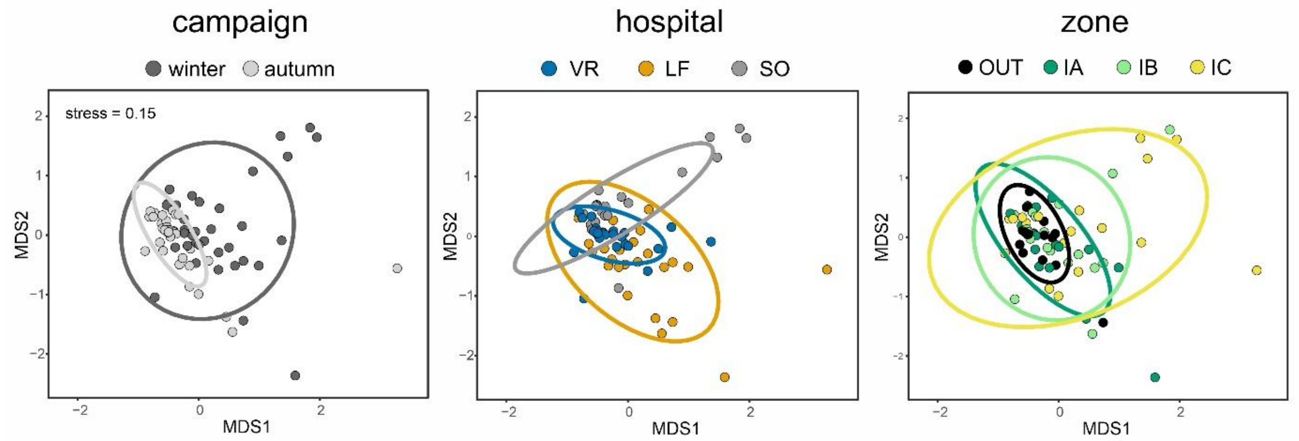

Figure S2. NMDS ordination plots showing the compositional variation of hospital mycobiomes from air samples (1,657 OTUs from 72 samples). Points represent air samples, and their colors indicate sampling campaigns (left), hospitals (middle) and hospital zones (right).

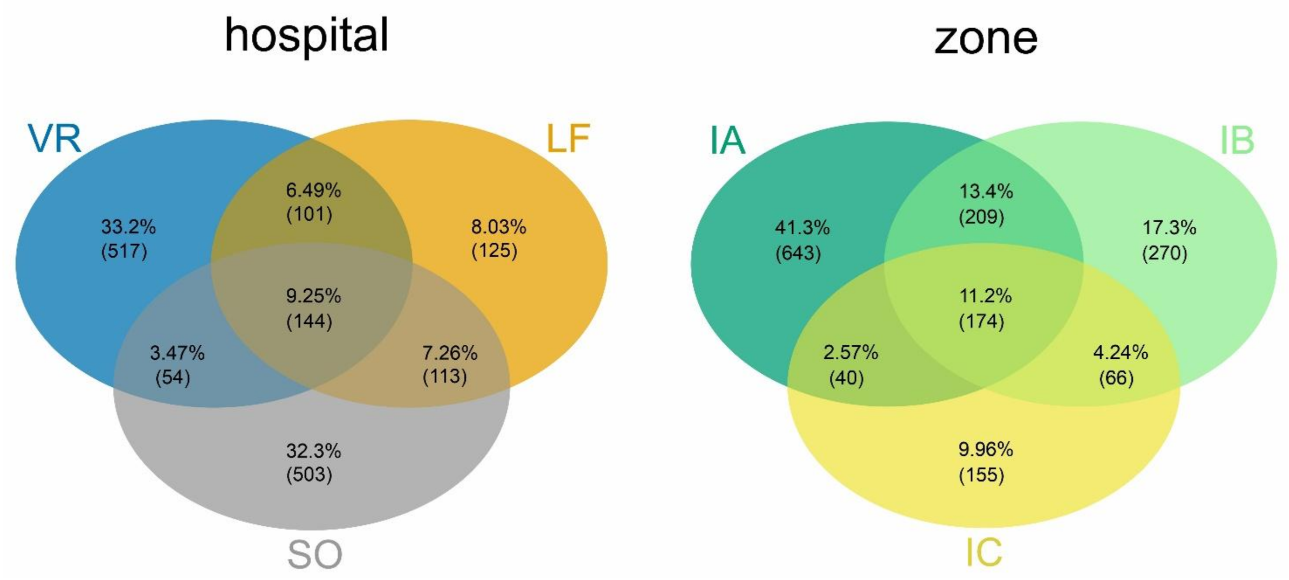

Figure S3. Venn diagrams showing the overlap of indoor mycobiomes (1,557 OTUs) for different hospitals and zones, as numbers and percentages of OTUs.

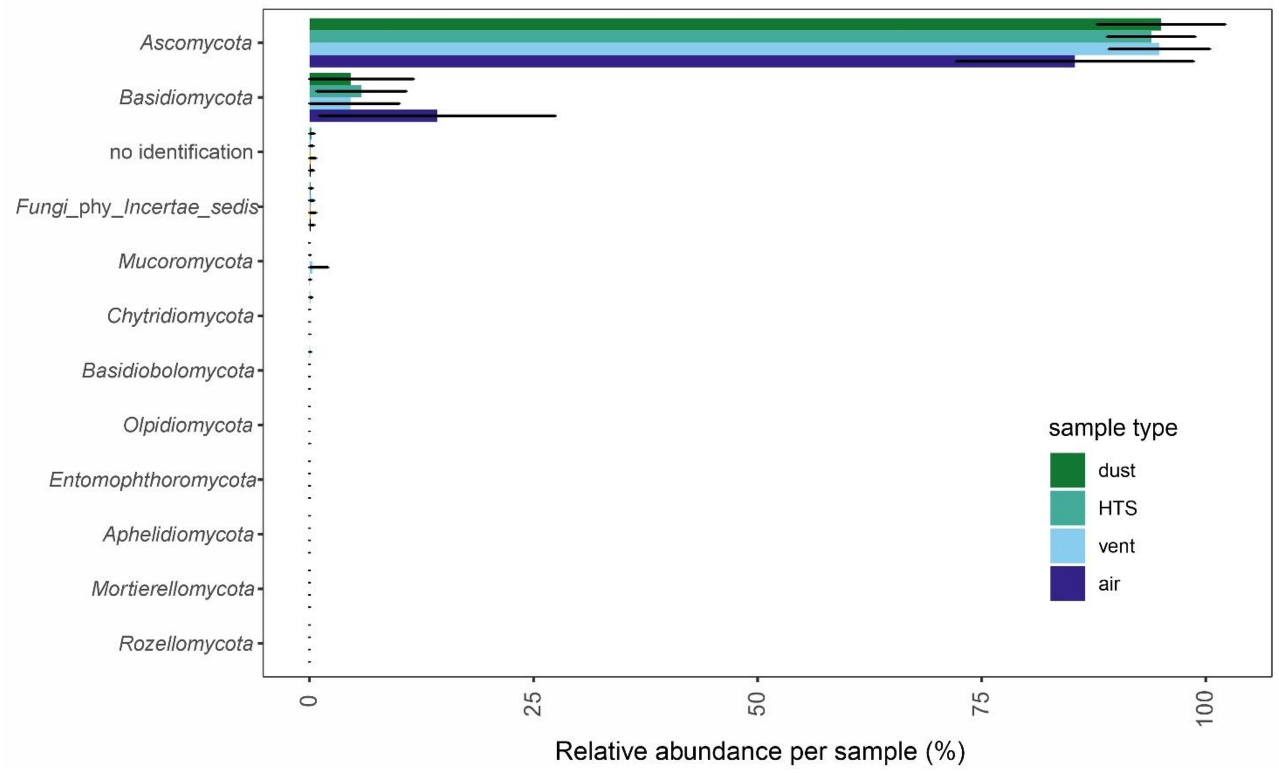

Figure S4. Fungal phyla identified in hospital environments detailing their relative abundances by sample types, calculated on the complete rarefied dataset after excluding soil samples (1,900 OTUs from 140 samples).

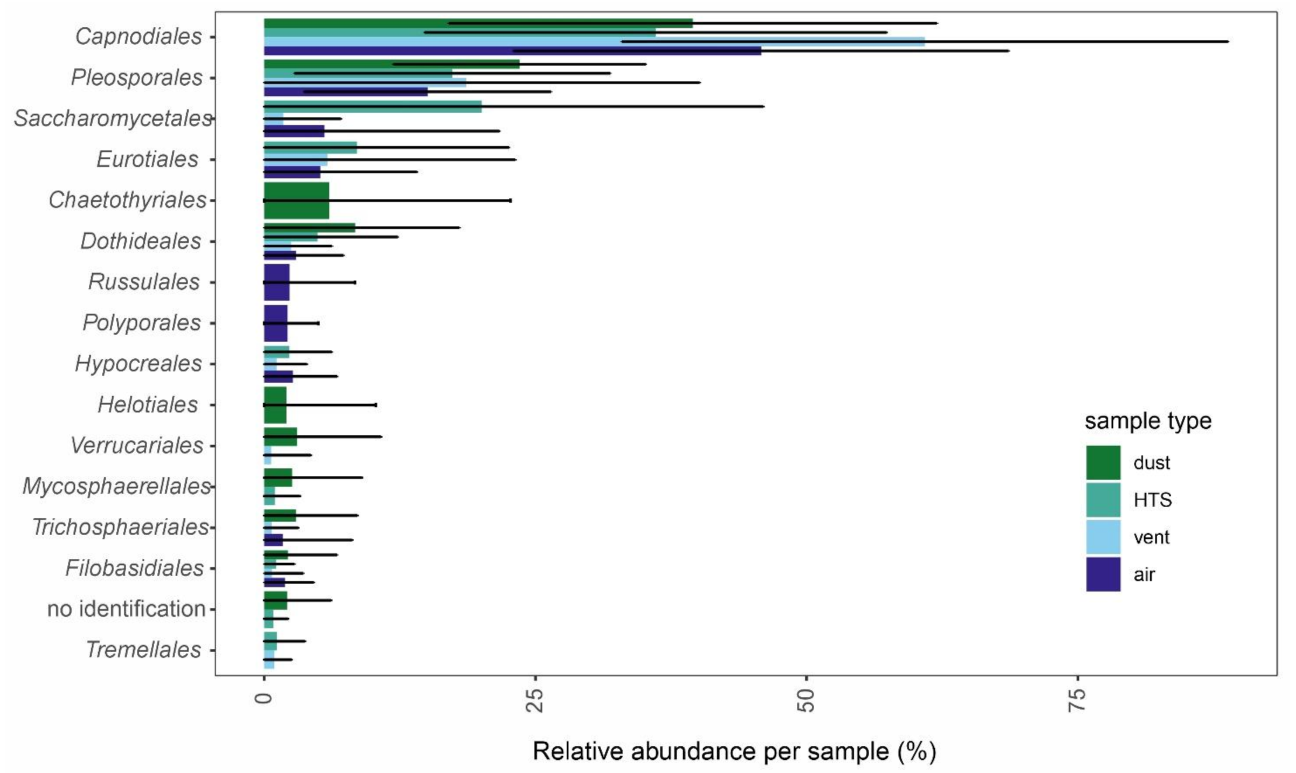

Figure S5. Most abundant fungal orders identified in hospital environments detailing their relative abundances by sample types, calculated on the complete rarefied dataset after excluding soil samples (1,900 OTUs from 140 samples).

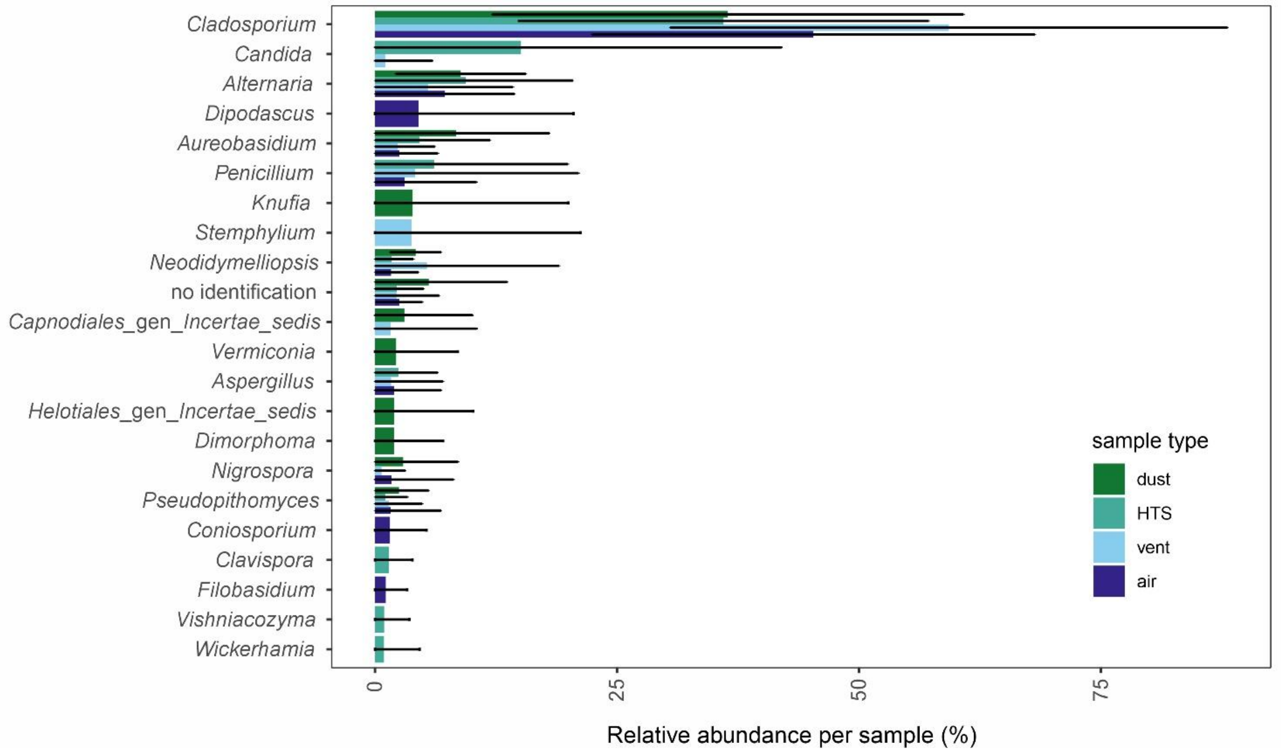

Figure S6. Most abundant fungal genera identified in hospital environments detailing their relative abundances by sample types, calculated on the complete rarefied dataset after excluding soil samples (1,900 OTUs from 140 samples).

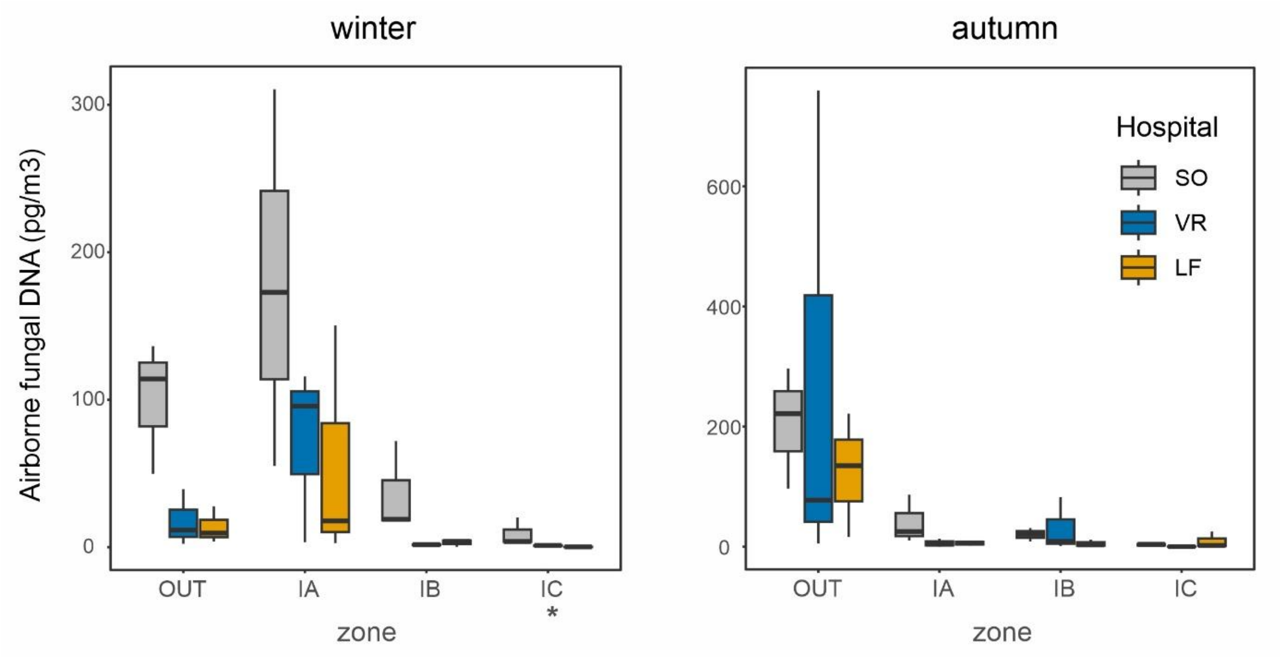

Figure S7. Airborne fungal DNA in different zones of the three study hospitals, in winter (left) and autumn (right), as assessed by quantitative PCR using universal fungal primers (NL1f and LS2r). Asterisk indicates the zone showing significant differences between hospitals according to Kruskal-Wallis test ($p \leq 0.05$). Lower and upper box boundaries are the 25th and 75th percentiles, respectively; line inside the box is the median; lower and upper lines are whiskers to minimum and maximum values, respectively.

Supplementary Table S1 (separate Excel file). Data for the 1,900 operational taxonomic units (OTUs) from the hospital mycobiomes, corresponding to the rarefied matrix excluding soil samples (*n* = 140 samples).
